# Multiple exon skipping strategies to by-pass dystrophin mutations

**DOI:** 10.1016/j.nmd.2011.10.007

**Published:** 2012-04

**Authors:** Carl F. Adkin, Penelope L. Meloni, Susan Fletcher, Abbie M. Adams, Francesco Muntoni, Brenda Wong, Steve D. Wilton

**Affiliations:** a Centre for Neuromuscular and Neurological Disorders, University of Western Australia, Perth, WA 6009, Australia; b The Dubowitz Neuromuscular Centre, UCL, Institute of Child Health, London, UK; c Department of Paediatrics, Cincinnati Children’s Hospital Medical Center, Cincinnati, OH, USA; d Department of Neurology, Cincinnati Children’s Hospital Medical Center, Cincinnati, OH, USA

**Keywords:** Dystrophin, Duchenne muscular dystrophy, Exon skipping, Antisense oligomers, Splice-switching, Personalised genetic therapy

## Abstract

Manipulation of dystrophin pre-mRNA processing offers the potential to overcome mutations in the dystrophin gene that would otherwise lead to Duchenne muscular dystrophy. Dystrophin mutations will require the removal of one or more exons to restore the reading frame and in some cases, multiple exon skipping strategies exist to restore dystrophin expression. However, for some small intra-exonic mutations, a third strategy, not applicable to whole exon deletions, may be possible. The removal of only one frame-shifting exon flanking the mutation-carrying exon may restore the reading frame and allow synthesis of a functional dystrophin isoform, providing that no premature termination codons are encountered. For these mutations, the removal of only one exon offers a simpler, cheaper and more feasible alternative approach to the dual exon skipping that would otherwise be considered. We present strategies to by-pass intra-exonic dystrophin mutations that clearly demonstrate the importance of tailoring exon skipping strategies to specific patient mutations.

## 1 Introduction

Antisense oligomer (AO) mediated exon skipping is a promising therapeutic approach for Duchenne muscular dystrophy (DMD), with rescue of functional dystrophin isoforms demonstrated in mouse and canine models of muscular dystrophy [1–3], including the severely affected utrophin:dystrophin deficient (*dko*) mouse [4]. Successful proof-of concept clinical studies using two different oligomer chemistries have been reported in DMD patients [5–8]. These trials have demonstrated that direct intramuscular injection or systemic delivery of two different oligomers, to DMD patients with amenable mutations, could excise dystrophin exon 51, thereby restoring the reading-frame and result in expression of correctly localised Becker muscular dystrophy (BMD)-like dystrophin isoforms.

Exon skipping is most commonly considered for reading-frame restoration around genomic whole exon deletions, as these represent the most common type of DMD mutation. Although there are two deletion hotspots in the *DMD* gene [9], other more subtle DMD mutations, including nonsense mutations, splice motif defects and intra-exonic insertions or deletions (indels) are spread across the gene. The nature and location of the mutation will dictate the splice switching strategy required to address different mutations, with *in silico* prediction tools facilitating exon skipping strategy selection [10]. It is generally assumed that the most appropriate strategy will also be the simplest, that is, excision of the minimum number of exons to restore the reading frame. However, other factors must be taken into account, including the efficiency of individual exon removal, and in cases where there are alternative options (Table 1), the predicted functionality of the induced dystrophin isoforms. For example, a frame-shifting deletion of exon 51 may be addressed by removal of either exon 50 or 52. If both exons can be removed with similar efficiency, then target selection should be determined by the functionality of the resultant protein. The removal of exons 51 and 52 would retain a portion of hinge 3, while excision of exons 50 and 51 will remove the entire hinge. It is yet to be determined which of these dystrophin isoforms would retain the greatest functionality, though patients have been documented with a deletion of exons 50 and 51, presenting with a mild BMD phenotype [11]. Studies are currently ongoing to assess dystrophin isoform function based upon exon boundaries [12] and structure of intact or partial spectrin repeats in the rod domain [13–15]. If there is no substantial difference between the two resulting isoforms, then, applicability to other mutation subtypes could be considered; skipping exon 50 or 52 could benefit 5.2% and 4.0% of DMD deletions, respectively [16]. Also, potential single nucleotide polymorphisms (SNPs) located within AO binding sites that may affect oligomer annealing must also be considered.

**Fig. 1:**
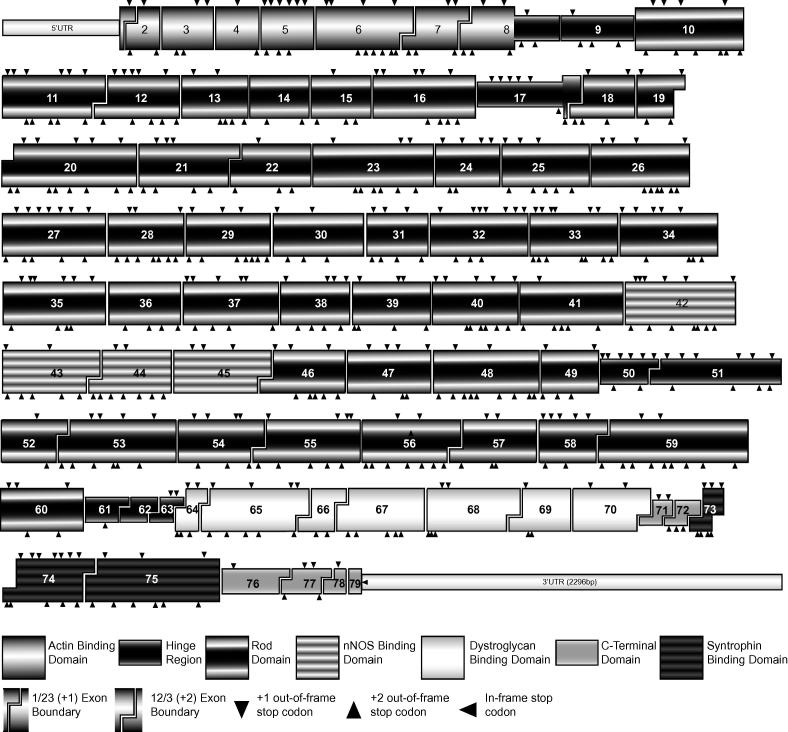
Exon arrangement and functional domains of the dystrophin transcript. Exons and functional domains are approximately to scale and in-frame/out of frame exon boundaries are indicated. Arrow heads on the top of the exon indicate locations of stop codons induced by a 1 base shift in the reading frame, stop codons induced by a 2 base shift are shown by an arrow head below the exon.

Thirty-nine of the seventy-seven “skippable” dystrophin exons (Fig. 1), if one excludes the first and last exon, can be removed individually without compromising the reading frame (Table 2). DMD-causing mutations confined to most of these in-frame exons are typically intra-exonic, and include nonsense mutations, splice site defects and small insertions/deletions (indels) that disrupt the reading frame, resulting in premature termination of translation. Consequently, these mutations, spread across approximately half of the gene could be removed from the mature dystrophin mRNA by single exon excision [1,17–19].

Intra-exonic mutations within frame-shifting exons will normally require two or more exons to be excised, the lesion-carrying exon and a flanking exon(s) to maintain the reading frame. Some such cases present alternative options in that either of the flanking exons may be excised (Table 1). However, particular mutations at or near splice sites present a third, simpler option where only one exon need be excluded to restore the reading frame. These selected mutations, typically located near the exon boundaries, may allow the mutated exon to remain in place while the flanking, frame-shifting exon is excised to restore the reading frame. The nature of the mutation creates a new reading frame, and provided that no stop codons are created between the lesion and the novel exon junction, these mutations can be addressed by single exon removal. This option offers a minimalist exon skipping strategy and maximises retained dystrophin coding sequence.

Although promising results have been reported for the Phase 2 exon 51 clinical trials using the morpholino oligomer AVI-4658 [8], unequivocal therapeutic benefits have yet to be demonstrated. Long-term safety and tolerability to the antisense oligomers must be demonstrated and exon skipping is likely to be implemented into the clinic in stages, and it is probable that dual exon skipping would be delayed until single exon skipping has been well established. The strategy reported here may expedite the application of AO induced exon skipping to selected DMD patients.

## 2 Materials and methods

### 2.1 Antisense oligomer design and synthesis

Splice switching AOs, designed to excise human dystrophin exons, have been described previously [20–22]. 2′-O-methyl modified bases on a phosphorothioate backbone (2OMeAOs) were prepared on an Expedite 8909 Nucleic acid synthesiser using the 1 μM thioate synthesis protocol. The compounds described here target normal dystrophin exons 19, 20, 21, 22, 50, 51 and 52 and are designed to anneal to splicing motifs at the intron/exon boundaries, as well as exonic splice enhancer (ESE) motifs predicted by the web based application, ESEfinder [23]. These AOs had been previously optimised and evaluated for inducing specific exon skipping after transfection into normal myogenic cells [21,22].

### 2.2 Primary human myoblast and fibroblast propagation

All patient biopsy samples were collected with informed consent and de-identified. The use of human tissue was approved by the University of Western Australia Human Ethics Committee (approval number RA/4/1/2295). Myogenic cells were propagated as previously described [24]. Fibroblast cultures were propagated from skin biopsies as previously described [25]. Prior to transfection with AOs, normal myogenic cells were plated at confluent density and allowed to differentiate for 1–3 days in low serum medium, prior to transfection. Fibroblast cultures were first transformed to a myogenic lineage with a MyoD expressing adenoviral vector (The Native Antigen Company, Oxford, UK) [26], at an MOI of 200 and allowed to differentiate for 2–3 days, once multinucleated myotubes were observed, prior to transfection.

### 2.3 Sequencing and splice site prediction

Genomic DNA was isolated using Trizol reagent as per the manufacturer’s instructions (Invitrogen, Melbourne, Australia). PCR amplicons were isolated for sequencing using the band stab protocol [27] and sequenced at the Australian Genome Research Facility (Perth node). Sequence analysis was performed using VectorNTI software (Invitrogen). Patient mutations are summarised in Table 3. Exonic splice enhancers sites found in normal and mutated *DMD* transcripts were predicted using ESEFinder3(c) [28]. Splice site scores were predicted using the alternative exon calculator tool from the Zhang Group at Cold Spring Harbour laboratories, http://www.rulai.cshl.edu/new_alt_exon_db2/HTML/score.html.

### 2.4 Antisense oligomer transfection and RT-PCR analysis

2OMe AOs were transfected into differentiated myotubes in Opti-MEM (Invitrogen) as cationic lipoplexes with Lipofectamine 2000 reagent at 1:1 w:w ratio, according to the manufacturer’s instructions (Invitrogen). Concentrations stated are total AO concentrations, where a cocktail of AOs is used; the concentration is the total of all AOs at 1:1 ratio. AOs used are listed in Supplementary Table 1. Transfected cells were incubated for 24–48 h before total RNA extraction using Trizol reagent according to the manufacturer’s instructions (Invitrogen) and RT-PCR was carried out as previously described by nested RT-PCR [29]. RT-PCR products were separated by agarose gel electrophoresis with 100 bp DNA ladder (Invitrogen). Amplification and sequencing primers used are listed in Supplementary Table 2. All transfection and RT-PCR analyses were carried out a minimum of two times.

## 3 Results

### 3.1 Confirming efficacy of AO cocktails in normal human myogenic cells

**Fig. 2:**
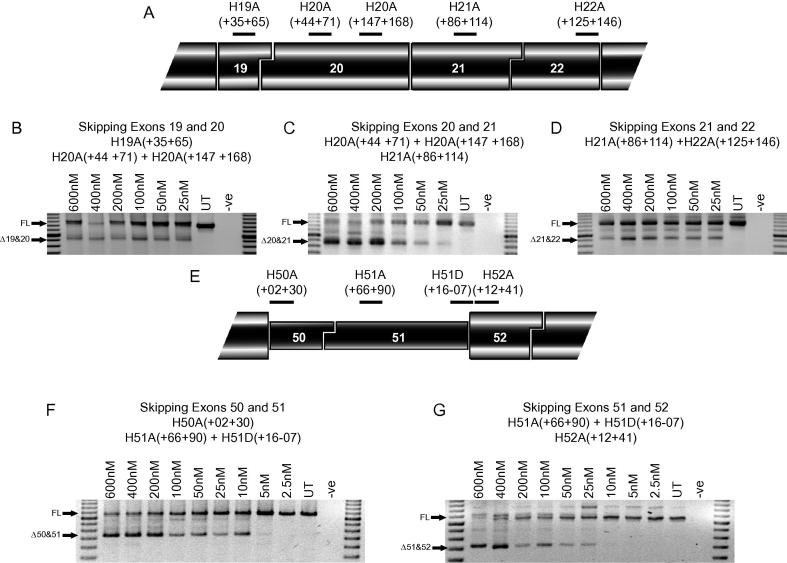
Skipping of exon pairs in normal human myogenic cultures. (A) Locations of AO annealing sites in human dystrophin exons 19, 20, 21 and 22. Nested RT-PCR across exons 17–25 showing skipping of pairs of exons with AO cocktails targeted to exons (B) 19&20, (C) 20&21, and (D) 21&22 at total AO concentrations of 600–25 nM. Marker is a 100 bp ladder. Full-length (FL) transcript is 1255 bp, Δ19&20 transcript is 925 bp, Δ20&21 transcript is 832 bp and Δ21&22 transcript is 928 bp. (E) Locations of AO annealing sites in human dystrophin exons 50, 51 and 52. Nested RT-PCR across exons 48–55 showing skipping of pairs of exons with AO cocktails targeted to exons (F) 50&51 and (G) 51&52 at total AO concentrations of 600–2.5 nM. Marker is a 100 bp ladder. Full length (FL) transcript is 1087 bp, Δ50&51 transcript is 745 bp and Δ51&52 transcript is 736 bp.

Before AOs were transfected into cells from DMD patients, their efficacy was confirmed in normal human myogenic cells (Fig. 2). Cocktails of AOs to skip exons 19&20, exons 20&21 and exons 21&22 showed that each pair of exons could be efficiently removed at the lowest concentration tested of 25 nM. There was little difference in efficacy between these cocktails, except exon 20&21 skipping was more effective at the higher concentrations of 200–600 nM. Skipping of exons 50 + 51 was induced at a concentration of 5 nM, and was marginally more effective than skipping exon 51&52, which could only be detected in cells treated with 25 nM of AO and not at lower concentrations.

### 3.2 Alternative strategies to address intra-exonic mutations

**Supplementary Figure 1:**
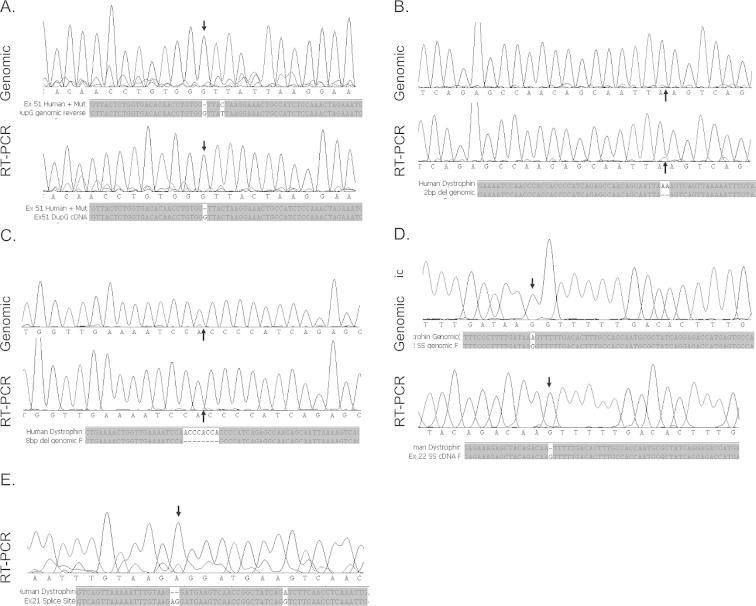
Exonic splice enhancers (ESEs) in dystrophin exon 20 and 51 predicted by ESE finder 3. (A) ESEs in exon 20 with normal dystrophin sequence and with 8bp deletion and 2bp deletions. ESEs affected by the mutations are circled. (B) ESEs in exon 51 with normal and mutated (7348DupG) dystrophin sequence. No effect on ESEs was observed with this mutation.

**Supplementary Figure 2:**
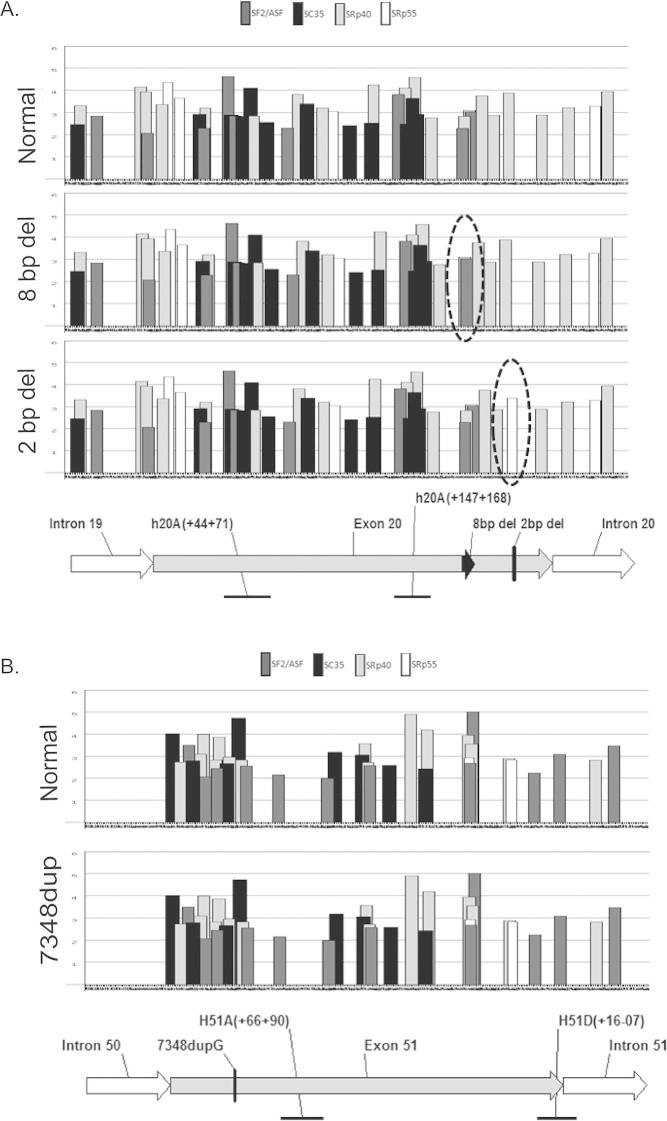
Exonic splice enhancers (ESEs) in dystrophin exon 20 and 51 predicted by ESE finder 3. (A) ESEs in exon 20 with normal dystrophin sequence and with 8bp deletion and 2bp deletions. ESEs affected by the mutations are circled. (B) ESEs in exon 51 with normal and mutated (7348DupG) dystrophin sequence. No effect on ESEs was observed with this mutation.

Here we present three examples of disease-causing intra-exonic mutations in the *DMD* gene. One in exon 51, (Patient A, c7348dupG) a single G duplication and two in exon 20: a 2 bp deletion (Patient B, c2601-2602delAA) and an 8 bp deletion (Patient C, c2568-2575delACCCACCA), all of which induce a reading frame-shift and dystrophin protein truncation, resulting in a DMD phenotype. The mutations were confirmed by sequencing genomic DNA and RT-PCR amplicons (Supplementary Fig. 1) and the affect of the mutations on ESEs was assessed (Supplementary Fig. 2). There are two alternative strategies available to restore the reading-frame in each of these mutations. Mutations in exon 51 can be corrected by skipping exons 50&51 or exons 51&52, mutations in exon 20 are correctable by skipping exons 19&20 or 20&21.

**Fig. 3:**
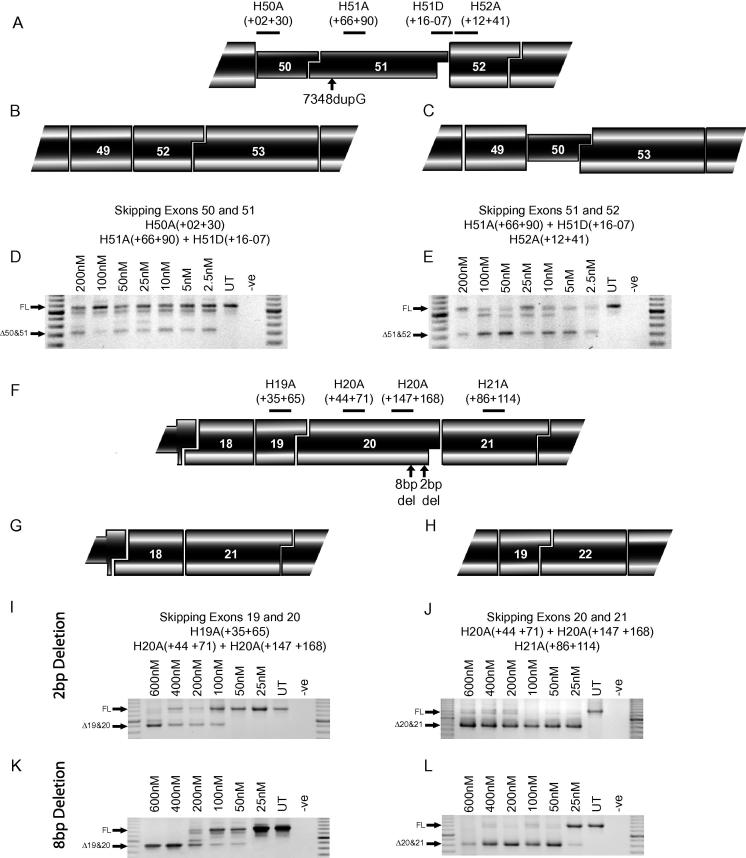
Alternative strategies for intra-exonic mutations. (A) Locations of AO annealing sites in human dystrophin exons 50, 51 and 52, the location of the 7348DupG mutation and the two options to correct a frame-shifting mutation in exon 51 are indicated: (B) skipping exons 50&51 and (C) skipping exons 51&52. Nested RT-PCR across exons 48–55 showing skipping of exons (D) 50&51 and (E) 51&52 in MyoD transformed patient fibroblasts. Full-length (FL) transcript is 1088 bp, Δ50&51 transcript is 745 bp and Δ51&52 transcript is 736 bp. (F) Locations of AO annealing sites in human dystrophin exons 19, 20 and 21, and the location of the 2 and 8 base deletions. Two options to correct these frame-shifting mutations in exon 20: (G) skipping exons 19&20 and (H) skipping exons 20&21. Nested RT-PCR across exons 17–25 shows skipping of exons 19&20 in (I) 2 base deletion cells and (K) 8 base deletion cells, and skipping of exons 20&21 in (J) in 2 base deletion cells and (L) 8 base deletion cells. Marker is a 100 bp ladder. Full-length (FL) transcript is 1255 bp, Δ19&20 transcript is 925 bp, Δ20&21 transcript is 832 bp and Δ21&22 transcript is 928 bp.

In MyoD transformed fibroblasts carrying the exon 51 duplication G, cocktails of oligomers targeted to exon 50&51 and 51&52 induced skipping of both exons at the lowest concentration of 2.5 nM (Fig. 3). The exon 51&52 cocktail appeared marginally more effective, demonstrated by the reduced level of full-length transcript, compared to the 50&51 cocktail (Fig. 3D and E).

The consequences of the small intra-exonic deletion (2 and 8 bases) are illustrated in Fig. 3F, as are the two dual exon skipping strategies to restore the reading frame (Fig. 3G and H). In the cells carrying the 2 bp deletion in exon 20, the cocktail targeting exons 19&20 induced skipping of the two exons at 100 nM, but skipping could not be detected in the cells treated at lower concentrations (Fig. 3I). The cocktail targeting exons 20&21 showed highly efficient skipping, close to 100% of both exons were excised at the lowest transfection concentration of 25 nM (Fig. 3J). In the cells with the 8 bp deletion, skipping of exons 19&20 was 100% after AO transfection at 400–600 nM, however in cells transfected with 200 nM of AO, skipping of both exons was observed in 60% of transcripts, with intermediate transcripts, lacking exon 19 or 20 only, also detectable (Fig. 3K). Levels of exon skipping were reduced to approximately 10–20% after transfection at 50 nM of AO, and undetectable at 25 nM. Exon 20&21 skipping was also highly efficient and could be induced to approximately 90% by transfection at 50–600 nM, and a low level of skipping could be detected at 25 nM (Fig. 3L).

### 3.3 Personalised strategies for selected splice-site mutations

**Fig. 4:**
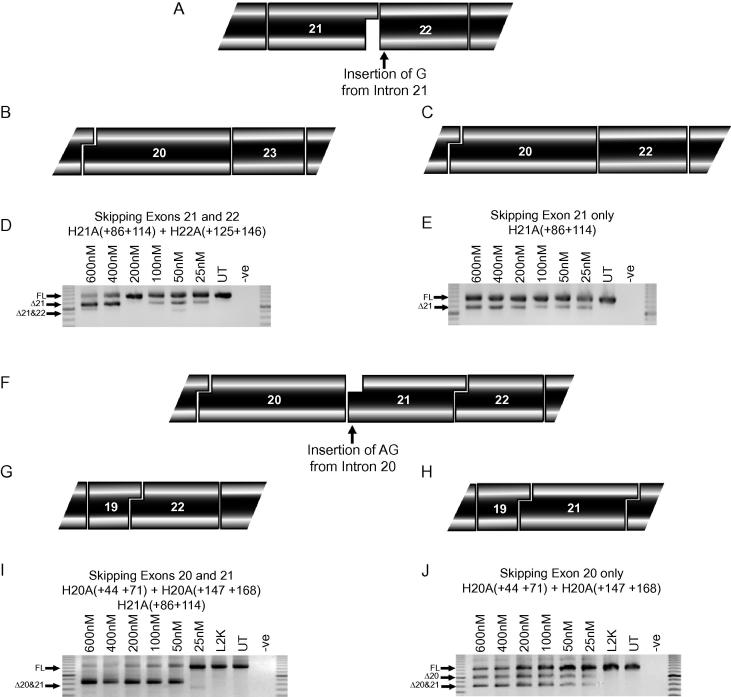
Alternative strategies for exon 21 and 22 splice site mutations. (A) A single A>G mutation at the exon 22 acceptor splice site induces retention of a single G nucleotide from intron 21. The reading frame is restored by (B) excision of exons 21&22 or (C) excision of exon 21 only. Nested RT-PCR across exons 17–25 showing (D) skipping of exons 21&22 or (E) exon 21 only in MyoD transformed patient fibroblasts with total AO concentrations of 600–25 nM. (F) A single C>G mutation at the exon 21 acceptor splice site allows retention of 2 nucleotides from intron 20. The reading frame is restored by exclusion of (G) skipping of exons 20&21, or (H) exon 20 alone. Nested RT-PCR across exons 17–25 showing skipping of (I) exons 20&21 and (J) exon 20 only, at total AO concentrations of 600–25 nM. Marker is a 100 bp ladder. Full-length (FL) transcript is 1255 bp, Δ20 transcript is 1013 bp, Δ21 transcript is 1074 bp and Δ20&21 is 832 bp.

A DMD patient was identified with a point mutation (Patient D, c2804-2C>G) at the exon 22 acceptor splice site (Fig. 4A). This base change altered the normal splice site score from 1.3 to −9.7 and activated a cryptic splice site with a score of 2.4. The retention of the last G nucleotide from intron 21 shifted the reading frame to convert exon 22 into an in-frame exon. Employing conventional exon skipping strategies, a frame-shifting mutation involving exon 22 could be treated by the excision of exons 21&22 (Fig. 4B). However, in this case, the additional base at the beginning of exon 22 allows the removal of exon 21 only to restore the reading frame (Fig. 4C). The two potential strategies to address this mutation were tested in MyoD transformed fibroblasts from the patient. Targeting a cocktail of AOs to exons 21&22 showed that this pair of exons could not be effectively removed at the concentrations tested, and skipping of exon 21 only could be detected (Fig. 4D). Targeting exon 21 alone showed efficient removal of this exon at all concentrations tested (Fig. 4E).

Another patient diagnosed with DMD was found to carry a single base change (Patient E, c2623-3C>G), at the acceptor splice site of exon 21. This mutation changed the normal splice site score from 4.2 to −6.3, and created an alternative splice site with a score of 3.1. This induced retention of the last 2 AG nucleotides of intron 20 in the mature dystrophin mRNA (Fig. 4F). The two conventional exon-skipping strategies to remove the gene lesion and restore the reading frame involve excision of exons 20&21 (Fig. 4G) or 21&22, with an example of robust exon skipping of the former shown in Fig. 4I. As with the exon 22 mutation described earlier, removal of exons 21&22 was not efficient (data not shown). However, the retention of the last 2 nucleotides of intron 20 would off-set the frame-shifting loss of 2 bases if only exon 20 was excised, presenting a third potential option to restore the reading frame for this mutation (Fig. 4H). Surprisingly, targeting only exon 20 also induced transcripts missing exons 20&21, however these are in-frame (Fig. 4J).

## 4 Discussion

DMD is a serious muscle wasting condition for which there is no treatment. The application of splice switching oligomers to remove or correct frame-shifting mutations that would otherwise lead to premature termination of translation has progressed from a concept to clinical trials. Proof-of-concept has been demonstrated in animal models and recently in DMD patients, with completion of Phase I intramuscular studies and Phase I/IIa systemic studies with two different oligonucleotide analogues [5–8]. The DMD mutation profile and incidence will determine exon targets for subsequent splice switching clinical trials, and these studies must focus on single exon removal, due to the number of responsive patients and cost of safety and toxicology validation required for each new oligomer [30]. Dual exon skipping to restore dystrophin expression has been demonstrated in animal models [3,12,31,32] and is technically feasible, but will be further from the clinic. The targeting of two or more exons in human dystrophin has been reported by others using AO cocktails [22,29,33] or AOs joined by a linker.

DMD is regarded as an orphan disease, with an estimated 420,000 affected individuals worldwide [34]. However, when further classifying amenable DMD mutations into subclasses, we suggest that exon skipping should be regarded as a personalised genetic treatment. As such, any strategies to implement exon skipping should be considered on a case-by-case basis, and it is important that the simplest and most effective exon skipping strategy for each mutation be carefully considered. The factors that will determine the most suitable strategy and AO sequence include the following: Which AOs are the most efficient at excising the target exon(s) *in vitro*? Which strategy will produce the most functional truncated dystrophin isoform, determined by BMD patient data and transient inducible mouse model [12]? What are the potential off-target effects of the AO treatment, including effects related to AO chemistry and sequence dependant effects? How many other patients could potentially be treated by skipping the combination of exons [30]?

This study reports DMD mutations that can be addressed by multiple strategies to restore the reading frame and facilitate translation of a BMD-like dystrophin isoform. This includes intra-exonic mutations in exons 51 and 20. In these cases, the mutated exon must be excised with either flanking exon. The mutation in exon 51, was marginally more efficiently addressed, at least *in vitro*, by skipping of exon 51 + 52. This is in contrast to the result in normal myogenic cells where exons 50 + 51 were more efficiently removed. ESE analysis did not show any alterations to ESEs as a result of the mutation, yet this is not conclusive evidence that the exon 51 disease-causing variant does not influence splicing. We previously demonstrated that a nonsense mutation in dystrophin exon 17 did not alter the efficiency of oligomers targeted to excise exon 17, but did influence exon 18 skipping [29]. This suggests subtle cross communication between exons during splicing, and further emphasises the need for empirical AO testing in patient-derived cells. In terms of the effect on the functionality of the resulting dystrophin isoform, it is not yet clear if dystrophin lacking exon 50&51, and therefore the whole of hinge 3 will be more functional than dystrophin lacking exons 51&52 where part of hinge 3 is retained. It has been reported that in-frame dystrophin deletions involving hinge 3 have a milder phenotype compared with other shorter deletions that do not involve that domain [35].

In cells from the patients with small intra-exonic deletions in exon, exons 20&21 were consistently removed more efficiently than exons 19&20. This is in agreement with the results obtained in normal myogenic cells. Both these mutations did cause small changes in the predicted ESE sites, but this does not appear to have affected the efficiency of the AO cocktails. Again, the effect on the functionality of the resultant dystrophin must be considered. In an AO induced transient mouse model, exons 19&20 could be removed from 100% of transcripts in the mouse diaphragm, with no detectable detrimental effects on the muscle phenotype [12]. The Leiden DMD database contains 2 patients lacking exons 19&20 [36,37], both are classified as DMD however the mutation screening was conducted using multiplex PCR and may not be fully informative [38]. One patient with a deletion of exons 20&21 is classified as BMD (DMD mutation database on http://www.dmd.nl), although detailed clinical data is not publicly available for these patients.

We also report on single base changes that alter splicing at particular exon boundaries and present unique opportunities for simpler, alternative exon skipping strategies. In these cases, additional factors should be considered, other than which is the most efficient strategy and which results in the most functional dystrophin isoform. We have demonstrated that for two splice site mutations an opportunity exists to restore the reading frame by excision of a single exon, rather than the two exons suggest by conventional exon skipping strategies. For the exon 22 acceptor splice site mutation, the results demonstrate that skipping both exons 21&22 is inefficient in the patient’s cells, while skipping exon 21 alone is a much simpler strategy and is more efficient *in vitro* in patient cells. Some large in-frame *DMD* deletions are associated with a mild phenotype, however this is greatly dependent upon the location of the *DMD* mutation [39,40]. In general, the loss of a smaller region of the dystrophin protein should confer greater functionality, and therefore a milder phenotype. In addition to the loss of exon 21 sequence, this strategy results in an isoleucine to valine amino acid substitution. Both amino acids possess large, non-polar, strongly hydrophobic side chains; hence the substitution could be predicted to have a minimal effect on the structure and function of the protein.

In normal myoblasts, skipping exon 20&21 or exon 21&22 was comparable, with the 20&21 cocktail proving marginally more efficient. In MyoD converted fibroblasts from the patient carrying the exon 21 splice site mutation, exon 20&21 were effectively removed by an AO cocktail. Exon 20 was also effectively excised on its own. In this case the inserted bases at the beginning of exon 21 (AG) are identical to the last two bases of exon 20, so excision of exon 20 does not alter the remaining amino acid sequence. Unexpectedly, it appears that the mutation facilitates skipping of exon 21 when exon 20 only is targeted, possibly because the exon 21 acceptor splice site is compromised. However, as both these transcripts are in-frame and allow dystrophin translation, targeting exon 20 only remains a viable option for this patient, despite this unexpected effect on exon selection.

We propose a set of guidelines to be followed when dealing with splice site mutations that may be amenable to splice manipulation. The “minimalist” exon skipping strategies will be more applicable to mutations that occur near exon boundaries, and to minimise the possibility of encountering in-frame stop codons, will be very dependent on the precise position and nature of the mutation. Therefore, careful analysis of the effect of the mutation on splicing of the exon is required and it must also be determined if the mutation affects the AO binding sites. Many exons are most efficiently removed by an AO targeted near the 3′ acceptor splice site and mutations in this region would therefore disrupt AO binding, and it may be necessary to use sub-optimal AOs that avoid the mutation, or design additional sequences specific to the mutation. Once these considerations have been taken into account, the other factors that impact upon the optimal strategy; efficient removal of the target exon, functionality of the resultant dystrophin, potential off-target effects, applicability to other DMD patients and cost-benefit analysis of the alternative strategies must be considered, before the optimal strategy is selected.

## 5 Conclusion

As splice switching oligomer therapies progress through early clinical studies, it will be necessary to demonstrate unequivocal benefits in response to the treatment and then expedite application to the widest possible cohort of DMD patients. We report DMD mutations that present multiple exon skipping strategies to correct the reading frame. In addition, we have shown that splice site mutations can present unique opportunities that reduce the number of exons that must be excised, producing a simpler, more efficient and less costly strategy that could potentially result in a more functional dystrophin. This highlights the need to carefully characterise all DMD mutations to determine if they are suitable for treatment by exon skipping, and detailed investigation may be required to determine which will be the optimal strategy for each patient. This strengthens the case for splice-switching oligomers as a personalised genetic treatment for DMD, and emphasises aspects that should be considered as this therapy becomes available to amenable patients.

## Figures and Tables

**Table 1:** Intra-exonic mutations in frame-shifting exons in the *DMD* gene that could respond to alternative exon skipping strategies.

Intra-exonic defect	Strategy 1	Strategy 2
Mutation in exon 7	Skip 6 + 7 + 8	Skip 2–7
Mutation in exon 20	Skip 19 + 20	Skip 20 + 21
Mutation in exon 44	Skip 43 + 44	Skip 44 + 45
Mutation in exon 45	Skip 44 + 45	Skip 45 + 46
Mutation in exon 51	Skip 50 + 51	Skip 51 + 52
Mutation in exon 52	Skip 51 + 52	Skip 52 + 53
Mutation in exon 55	Skip 54 + 55	Skip 55 + 56
Mutation in exon 56	Skip 55 + 56	Skip 56 + 57
Mutation in exon 63	Skip 62 + 63	Skip 63 + 64 + 65
Mutation in exon 66	Skip 65 + 66	Skip 66 + 67 + 68
Mutation in exon 68	Skip 68 + 69	Skip 69 + 70

**Table 2:** In-frame exon blocks that may be removed from the dystrophin transcript that may be involved in by-passing protein truncating mutations.

In-frame exon combinations	Dystrophin exons
Single	3, 4, 5, 9, 10, 13, 14, 15, 16, 23–42, 47, 48, 49, 60, 64, 71, 72, 73, 74, 77
Two exons	11–12, 17–18, 19–20, 20–21, 21–22, 43–44, 44–45, 45–46, 50–51, 51–52, 52–53, 54–55, 55–56, 56–57, 58–59, 62–63, 65–66, 68–69, 69–70
Three exons	6–8, 59–61, 62–64, 63–65, 64–66, 66–68, 76–78
Larger in-frame blocks	Deletion of exon 2 restored by 3–7 skipping, deletion of 75 restored by skipping 70–75

**Table 3:** DMD patient mutations compared to human dystrophin mRNA sequence (NM_000109.3) and genomic sequence (NG_012232.1).

Patient sample	Mutation	Location	Result
A	c7348dupG	Exon 51 – Intra exonic	Frame shift
B	c2601-2602delAA	Exon 20 – Intra exonic	Frame shift
C	c2568-2575delACCCACCA	Exon 20 – Intra exonic	Frame shift
D	c2804-2C>G	Exon 22 – Acceptor splice site	1 bp of intron 21 retained
E	c2623-3C>G	Exon 21 – Acceptor splice site	2 bp of intron 20 retained

## Appendix A Supplementary data

Supplementary Table 1 List of antisense oligomers used. All oligomers were used as 20MeAOs.

Supplementary Table 2 List of primers used for nested RT-PCR and sequencing reactions.
